# Deep learning methods for 3D tracking of fish in challenging underwater conditions for future perception in autonomous underwater vehicles

**DOI:** 10.3389/frobt.2025.1628213

**Published:** 2025-10-17

**Authors:** Martin Føre, Emilia May O’Brien, Eleni Kelasidi

**Affiliations:** 1 Department of Engineering Cybernetics, Norwegian University of Science and Technology, Trondheim, Norway; 2 Department of Aquaculture, SINTEF Ocean AS, Trondheim, Norway; 3 Department of Mechanical and Industrial Engineering, Norwegian University of Science and Technology, Trondheim, Norway

**Keywords:** aquaculture, fish tracking, challenging optical conditions, perception in underwater robotics, deep learning

## Abstract

Due to their utility in replacing workers in tasks unsuitable for humans, unmanned underwater vehicles (UUVs) have become increasingly common tools in the fish farming industry. However, earlier studies and anecdotal evidence from farmers imply that farmed fish tend to move away from and avoid intrusive objects such as vehicles that are deployed and operated inside net pens. Such responses could imply a discomfort associated with the intrusive objects, which, in turn, can lead to stress and impaired welfare in the fish. To prevent this, vehicles and their control systems should be designed to automatically adjust operations when they perceive that they are repelling the fish. A necessary first step in this direction is to develop on-vehicle observation systems for assessing object/vehicle–fish distances in real-time settings that can provide inputs to the control algorithms. Due to their small size and low weight, modern cameras are ideal for this purpose. Moreover, the ongoing rapid developments within deep learning methods are enabling the use of increasingly sophisticated methods for analyzing footage from cameras. To explore this potential, we developed three new pipelines for the automated assessment of fish–camera distances in video and images. These methods were complemented using a recently published method, yielding four pipelines in total, namely, *SegmentDepth*, *BBoxDepth*, and *SuperGlue* that were based on stereo-vision and *DepthAnything* that was monocular. The overall performance was investigated using field data by comparing the fish–object distances obtained from the methods with those measured using a sonar. The four methods were then benchmarked by comparing the number of objects detected and the quality and overall accuracy of the stereo matches (only stereo-based methods). *SegmentDepth*, *DepthAnything*, and *SuperGlue* performed well in comparison with the sonar data, yielding mean absolute errors (MAE) of 0.205 m (95% CI: 0.050–0.360), 0.412 m (95% CI: 0.148–0.676), and 0.187 m (95% CI: 0.073–0.300), respectively, and were integrated into the Robot Operating System (ROS2) framework to enable real-time application in fish behavior identification and the control of robotic vehicles such as UUVs.

## 1 Introduction

Finfish aquaculture is a food production sector that that significantly contributes to aquatic proteins (FAO, 2024). Although intensive fish production is an industrial success story, fish farms need to cope with persistent challenges associated with various aspects of the production, including fish welfare (Martos-Sitcha et al., 2020), personnel safety (Thorvaldsen et al., 2020), farm integrity (Berstad et al., 2004), efficient feeding (Zhou et al., 2018), and biofouling (Bannister et al., 2019). Moreover, there are current industrial drives toward moving production practices to more exposed areas, a trend that is likely to amplify such issues (Bjelland et al., 2015; 2024). Although these challenges have traditionally been handled manually, emerging trends such as precision farming concepts seek to improve the ability and efficacy of farm management using autonomous and robotic tools (Føre et al., 2018). As technological solutions required for this shift become more advanced and less costly, the number of initiatives in this direction is increasing (Kelasidi and Svendsen, 2022), ranging today from external monitoring (Chalkiadakis et al., 2017) to in-pen monitoring (Kelasidi et al., 2022) and autonomous net cleaning (Ohrem et al., 2020; Fu et al., 2024).

All robotic operations conducted within net pens entail introducing an object into the habitat of the fish. This may disturb the fish, thus potentially perturbing the production process. Although this type of disturbance is difficult to quantify, earlier studies and anecdotal observations by farmers have shown that farmed fish usually try to maintain a certain distance from such intrusive objects. The preferred distance kept from an object seems to depend on its properties, and a specific link between object size and color and fish–object distance has been identified (Marras and Porfiri, 2012; Kruusmaa et al., 2020). To explore these effects in more detail, Zhang et al. (2024) recently conducted a systematic study in which fish in a commercial salmon farm were exposed to objects of different sizes, shapes, and colors, keeping the rest of the experimental setup and object properties as constant as possible to isolate the impacts of each specific factor. Data were collected in that study using a sweeping sonar that scanned a horizontal plane (i.e., 360 ° ) around the object every 8 s. For each case, a measure of the fish–object distance was obtained by accumulating and averaging all scans over 1, 5, and 10 min periods and then finding the shortest distance between the inner perimeter of the resulting fish distribution and the object. The main findings of Zhang et al. (2024) were that the fish kept an average distance of 3.8 body lengths from intrusive objects, that their preferred distances scaled with the object size (i.e., greater distances to larger objects), and that they stayed farther away from yellow objects than from white objects.

This type of avoidance behavior could imply a degree of discomfort that can ultimately lead to impaired welfare or stress. It is, therefore, desirable to design future robotic solutions with the intent of minimizing their impact on the fish. This will include revising the physical appearance of the vehicles and devising control systems that allow for the adjustment of movements and actions based on observed fish responses. These control systems will depend on perception tools that can automatically assess vehicle–fish distances and provide these as inputs in the control loop. The sonar approach used by Zhang et al. (2024) had a minimum scan time of 8 s for each full 360 ° scan. This enables accurate assessment of steady-state responses toward static and stationary objects but is not fast enough to capture transient responses induced by, for example, vehicle movements, sound emissions (thrusters and motors), toggling of light sources, or similar stimuli. Since a proper perception tool for this purpose will need to capture both stationary and transient responses to provide reliable inputs to the control system, new observation methods that measure fish–object distances with a high time resolution are, therefore, needed to complement the established sonar-based solution. Other sonar solutions with higher update frequencies such as multibeam devices (Kristmundsson et al., 2023) and acoustic cameras (Zhou and Mizuno, 2024) could represent alternatives here. However, it would be interesting to explore the potential of using optical solutions for this purpose as they tend to cost and weigh less than acoustic systems, and most underwater vehicles are already equipped with cameras, alleviating the need to install new hardware.

Computer vision (CV) is a field that has seen explosive development over the recent years, much because of affordable hardware solutions and an increasing number of AI-based methods available for automatic processing of videos and images. Although most early uses of CV in aquaculture were aimed at processing fish after slaughter (Misimi et al., 2008), these methods are increasingly being applied to analyze live fish in net pens or tanks (Saberioon et al., 2017). Recent studies have sought to apply CV to the problem of assessing distances to fish from mobile camera platforms (Saad et al., 2024). So far, this research has resulted in a pipeline for automatically obtaining the distance between fish and vehicles based on stereo video inputs (Alvheim et al., 2025). This pipeline was built around a published method for stereo matching and tracking called *SuperGlue* (Sarlin et al., 2020) and was proven able to obtain distance outputs that were comparable to those acquired with the aforementioned sonar solution (Zhang et al., 2024). Although the results from *SuperGlue* were promising, there exist several other methods capable of assessing fish–vehicle distances. Robotic operations in aquaculture face challenges that are not so prevalent in most CV applications, including limited visibility due to high turbidity, occlusion by fish, and variable underwater lighting. In addition, it is necessary to account for biological factors such as species-specific morphology, rendering the perception and detection of fish more challenging, and that the vehicle may elicit avoidance behavior as described above, potentially compromising the ability to observe undisturbed fish. A suitable onboard fish–vehicle distance assessment method, therefore, needs to cope with these and other challenges endemic to underwater use in addition to being able to run in real time onboard the robotic platform. It is thus prudent to compare the performance of several possible approaches to ensure that further work on developing a robot perception tool is based on the methods best able to balance the accuracy and robustness in fish–vehicle distance assessments and real-time operation capabilities.

The present study sought to compare the established *SuperGlue* pipeline with other stereo video and monocular (i.e., requiring only one video stream) methods for assessing depths in 2D images. Two alternative stereo methods (*SegmentDepth* and *BBoxDepth*) and one monocular method (*DepthAnything*) were chosen for this purpose. New pipelines similar to that of *SuperGlue* were developed for all three methods. The *SegmentDepth* pipeline required new modules for segmenting and stereo association of segmented masks; *BBoxDepth* required a module for stereo association through bounding box matching, while *DepthAnything* was implemented as a single module providing 3D points directly based on depth maps. The stereo-based pipelines were set up to track the caudal fins of the fish as this is a feature in farmed fish that is easy to distinguish and has lower potential for false positives than, for example, the entire fish body. To evaluate their ability to assess fish–object distances, the four methods were applied to selected video data collected from the field study described by Zhang et al. (2024). The results were then compared to assess the similarities and differences between the methods in estimating the depth in 2D images and tracking the trajectory of keypoints in the image streams in 3D. Distances obtained with the different pipelines were then validated by comparing the values obtained with the videos for each case in Zhang et al. (2024) with the corresponding distances previously obtained with sonar. The pipelines were then subjected to more detailed analyses comparing their ability to detect and track objects (i.e., caudal fins/fish) with the quality of the stereo matches (only for the stereo-based methods). The best performing pipelines were implemented in the Robot Operating System (ROS2) to ensure their compatibility with future implementation in robot- or vehicle-borne systems. A proper test of their real-time capabilities would require their implementation in the hardware of an actual robotic system. However, it is useful to first evaluate the publication frequency of the ROS2 implementation when running on a conventional desktop computer. Since the hardware capabilities of most robotic platforms are lower than those of a desktop computer, this test provides a first indication of whether real-time operation of the methods is theoretically possible and, if not, where the performance needs to be improved to achieve this. The publication frequencies achieved by the ROS2 implementation were, therefore, evaluated against update frequencies assumed to be necessary for autonomous navigation purposes (5–10 Hz).

## 2 Materials and methods

### 2.1 Data acquisition

**FIGURE 1:**
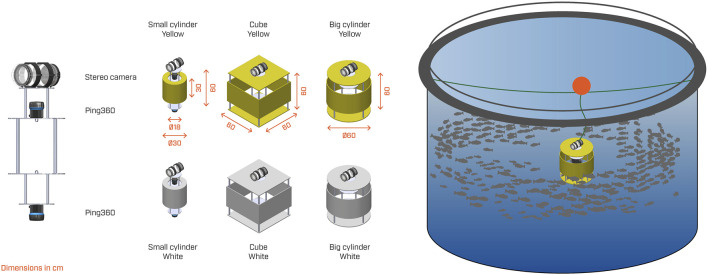
Experimental setup showing the center structure (left) carrying the stereo video system and the Ping360 sonar, the six different shape (small cylinder, cube, and big cylinder) and color (white and yellow) combinations of its exterior appearance (middle), and the deployment of the structure inside a commercial pen (right). Reproduced from Zhang et al. (2022).

This study used the same video footage as that used by Alvheim et al. (In review). Data were collected in a commercial-scale fish farm in September 2022 and featured fish with an average weight of 1,084 g (see Figure 1 for an overview of the full experimental setup). The fish were exposed to a structure that varied in shape (cylinder or cube), size (Ø30 × 30 cm, Ø60 × 60 cm, and 60 × 60 × 60 cm), and color (yellow or white), resulting in six cases (Figure 1). All cases are hereafter denoted with abbreviations: BY, big cylinder yellow; BW, big cylinder white; CY, cube yellow; CW, cube white; SY, small cylinder yellow; SW, small cylinder white. To capture the behavioral response of the fish, the structure was equipped with a custom-made stereo camera and Ping360 sonars (Blue Robotics Inc.) for collecting video footage and sonar data, respectively. The Ping360 sonars were set up with a range of 5 m, a 360 ° scan time of 8 s, and an angle step of 2 ° . The stereo camera was housed in a BlueRobotics 4″ watertight housing, featured two Lucid TRI032S-CC GigE Vision (Lucid Vision Labs Inc.) cameras attached to a 3D-printed bracket with a 42 mm baseline and was attached to the top of the structure (Figure 1). These cameras had a 3.14 MP Sony IMX265 color sensor, and each camera produced frames at a 1920×1200 pixel resolution. One of the cameras was configured as “master” and used hardware synchronization with the other camera. This was done by physically wiring the TTL digital signal output of the “master” camera to the TTL digital input of the other camera, thereby enabling triggering the exposure on the two cameras at the exact same time. Both cameras were set up with a fixed focal length, aperture, and exposure time, while digital auto-gain was used individually on each camera to control correct exposure at variable light intensities on different depths. The camera output was stored as an uncompressed AVI at 25 fps, with each frame being at the left and right images stitched together at 3840 × 1200 resolution. Stereo camera calibration was conducted prior to the experiments using underwater images of a chessboard plane with squares of known size (see Saad et al., 2024, for details on the calibration process for this camera setup).

The structure was placed 5 m from the net wall and at 8 m depth in all cases. For each case (i.e., shapes, colors, and sizes), six replicate videos of 12 min were recorded using the stereo video camera and the sonar, with the first and last minutes in each video being discarded to reduce the impact of transients on the analyses. The dataset for training, validation, and testing was built using every 50th frame from the left camera in the video collected when exposing the fish to the small yellow cylinder. This resulted in 686 images, which were labeled and made into a dataset (60% training, 20% validation, and 20% test) using Python and OpenCV, with annotation performed through CVAT. To reset the situation between the replicates, the structure was moved for approximately 25 s between repetitions. Except four replicates for CW where cleaner fish obstructed the view, all replicates were used in the analyses.

### 2.2 Implementation and development environment

The four processing pipelines were originally designed, developed, and implemented in a Python environment running on a desktop computer. These pipelines were used for offline analyses of the data from the field study to assess their respective abilities in scoping the depth in underwater images. However, to allow easier integration with underwater vehicles at a future point in time, the pipelines were also ported to a ROS2 environment. The ROS2 implementation featured only those pipelines deemed sufficiently accurate and efficient for use in real situations and was used in runtime analyses.

### 2.3 Processing pipelines

**FIGURE 2:**
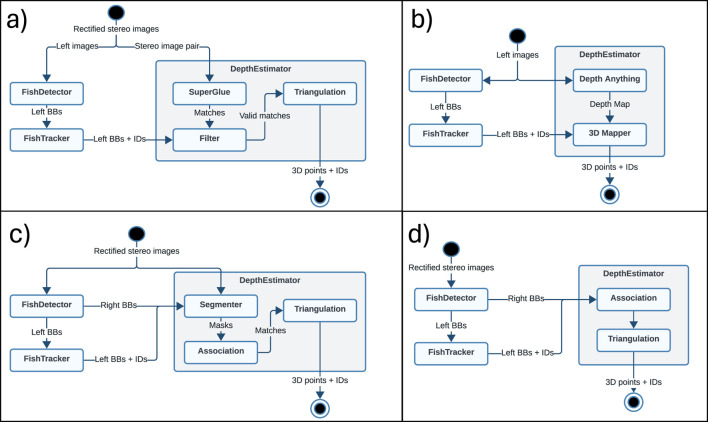
The four pipelines developed in this study, including the common elements (*FishDetector* and *FishTracker*) and components specific for each pipeline. **(a)** *SuperGlue* pipeline **(b)** *DepthAnything* pipeline, **(c)** *SegmentDepth* pipeline, and **(d)** *BBoxDepth* pipeline.

The pipelines developed and evaluated will hereafter be referred to as *SuperGlue*, *DepthAnything*, *SegmentDepth*, and *BBoxDepth*. Although all four pipelines had common modules for fish identification and tracking, each had their specific modules for handling the estimation of 3D positions of the tracked fish (Figure 2). *SuperGlue*, *SegmentDepth*, and *BBoxDepth* were designed for stereo video streams and thus used both video streams from the stereo video setup, while *DepthAnything* was a monocular method that only used video from the camera on the left. In the following, the different modules are discussed in more detail.

#### 2.3.1 Common modules for fish tracking

Two of the modules, *FishDetector* and *FishTracker*, were used in all pipelines, the first of which was responsible for detecting the caudal fins of individual fish and generating bounding boxes (BBs) to mark them as targets for tracking. To achieve this, the module was set up with a YOLOv8n (Jocher et al., 2023) model trained to detect caudal fins, which was developed and used by Alvheim et al. (In review). *FishDetector* was applied to both video streams in *SegmentDepth* and *BBoxDepth*, while it was only applied to the left camera when using *SuperGlue* and *DepthAnything*.

Using the outputs from *FishDetector*, the *FishTracker* module was set up with multiple objective tracking (MOT) capabilities to track all identified BBs, along with unique IDs, class names, and track confidences. After evaluating several candidates for MOT, the ByteTrack algorithm was chosen due to its reported accuracy, robustness, and real-time processing abilities (Zhang et al., 2022) using the setup parameters identified by Jakobsen (2023).

#### 2.3.2 *SuperGlue* pipeline

When using the *SuperGlue* pipeline (Figure 2a), both video streams were fed into the *SuperGlue* module, which used a CNN-based approach to detect and match features in stereo images (Sarlin et al., 2020). Since there exist no version of *SuperGlue* fine-tuned for underwater images, a model pretrained using outdoor images (the MegaDepth3 dataset) was used. Stereo matching with *SuperGlue* was done independently from fish identification, a feature that may produce several matches that are not related to fish. To prevent such no-fish matches from affecting the outcome, the left image stream was run through *FishDetector* and *FishTracker* to identify and track fish and mark these with BBs. Stereo matches identified by *SuperGlue* were then filtered (*Filter* module in Figure 2a), keeping only matched points with a confidence level higher than 0.1 that fell within identified BBs (i.e., being likely to be part of a fish), while the remaining matches were discarded. Each matching point was thereafter associated with the individual fish whose BB fell within on the left channel. To derive the 2D pixel positions to be used in the triangulation, the center point of the BB associated with the fish was first derived and used as the 2D position in the left image $\left(x_{l},y_{l}\right)$ . The 2D distance vector between this point and the average position of the matched points in the left image was then found, providing a measure of how far from the bounding box centroid this average position is. This vector was then added to the average position of the matched points in the right image, thereby estimating the position corresponding to the point $x_{l},y_{l}$ in the right image $\left(x_{r},y_{r}\right)$ . These two values were then triangulated to find the 3D position of the BB (and hence caudal fin) relative to the camera ( X , Y, and Z ) by applying Equation 1.

$$X=Z\cdot \frac{u-c_{x}}{f_{x}},\;Y=Z\cdot \frac{v-c_{y}}{f_{y}},\;Z=b\frac{f}{x_{l}-x_{r}}$$ (1)

Here, u and v are the horizontal and vertical 2D pixel coordinates, respectively; $c_{x}$ and $c_{y}$ are the center points of the 2D pixel image; $x_{l}-x_{r}$ is the disparity (pixels) ; b is the baseline (mm) ; and f is the focal length (mm) , $f_{x}$, and $f_{y}$, being the x and y components of the focal length.

#### 2.3.3 *DepthAnything* pipeline

Since *DepthAnything* is monocular, it was set up to use images only from the left camera. The *DepthAnything* framework offers three models of varying sizes for relative depth estimation (Yang et al., 2024). Although these models have different compromises between accuracy and speed, only the largest (and slowest) model has been tuned for metric depth estimation. Fine-tuning was done using the ZoeDepth code base, by initializing the *DepthAnything* model as an encoder training based on metric depth maps. Since there exist no underwater depth map data to train the model or pre-trained version using such data, the model used in this study was a downloaded version trained on data obtained in air indoors (the NYU-Depth v2 dataset).

During operation, the *DepthAnything* model first estimates a metric depth map for the entire picture frame (*DepthAnything* module in Figure 2b). The central pixel coordinates within each tracked BB received from *FishTracker* are then used to retrieve the depth of that pixel from the map, representing the distance to the tracked fish (*3D Mapper* module in Figure 2b).

#### 2.3.4 *SegmentDepth* pipeline

Segmentation models contain more information about the detected object than a bounding box. It is thus plausible that segmentation-based models can identify the same points of interest in both video streams more accurately than methods based on bounding box matching, thereby obtaining more accurate 3D positions. This was the motivation for setting up the *SegmentDepth* pipeline that included a segmentation model in the workflow, in addition to stereo matching and triangulation. Unlike for *SuperGlue*, the image streams from both right and left cameras were run through *FishDetector* to identify fish, with *FishTracker* also tracking the fish from the left camera. BBs detected in both camera streams were then fed into the segmentation method (*Segmenter* box in Figure 2c), which segmented the image within the boxes returning a segmentation mask. Although this segmentation should ideally be done using a model specifically trained to detect and segment caudal fins on fish, this would require a large training dataset of images where the caudal fins are already manually segmented. Since no such public datasets exist, a generic pre-trained, promptable segmentation model was used instead. The Segment Anything Model (SAM) ViT-B published by Meta (Kirillov et al., 2023) was chosen for this purpose as it has a relatively light-weight architecture, making it less computationally demanding than more complex models while still being more accurate than less complex models. Resulting masks from the two streams are then correlated (*Association* box in figure) using an assignment cost matrix quantifying overlap between the masks using IOU calculations, thus identifying matching cases for 3D-positioning. The centroids of the masks were found by averaging all their pixel coordinates and then subjected to triangulation (Equation 1) to yield the 3D positions.

#### 2.3.5 *BBoxDepth* pipeline

Instead of identifying all features that are matchable between left and right camera streams (as in *SuperGlue*) or correlating post-segmentation masks between the two streams (as in *SegmentDepth*), the *BBoxDepth* pipeline was based on directly matching the BBs provided by *FishTracker* and *FishDetector*. The motivation behind this approach was to test a less complex method that is likely to be less computationally demanding than the other stereo matching methods, albeit at a cost of being less accurate. Matching was done by calculating a similar assignment cost matrix for BB pairs as that used for masks by *SegmentDepth* (*Association* in Figure 2d). The boxes were optimally assigned using the Hungarian algorithm, before the triangulation routine (Equation 1) was run for the center points of the matched BBs to yield a 3D position.

### 2.4 ROS2 pipelines

Although the *DepthAnything*, *SuperGlue*, and *SegmentDepth* pipelines were all implemented in ROS2, *BBoxDepth* was considered less accurate, although otherwise comparable to *SegmentDepth*, and was therefore not included in the ROS2 implementation. The first two implemented pipelines were directly ported from the original Python code, while *SegmentDepth* required modification as initial testing proved that the approach to matching segmentation masks was too computationally intensive for real-time operation. This resulted in an alternative pipeline adapted to ROS2.

#### 2.4.1 Modified *SegmentDepth* pipeline

**FIGURE 3:**
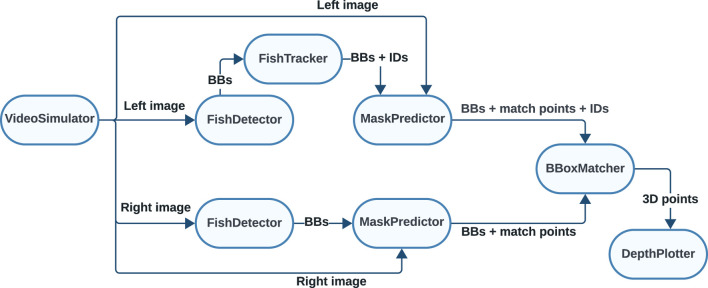
Modified *SegmentDepth* pipeline implemented in ROS2.

The modified *SegmentDepth* pipeline featured a new module called *BBoxMatcher*, which was inserted into the association step in the pipeline. This module did the stereo matching based on IOU using the bounding boxes instead of matching the segmentation masks as in the original *SegmentDepth* pipeline (Figure 3).

Although this meant that stereo matching was made based on the BBs, the segmentation masks were still found through SAM (the *MaskPredictor* module in Figure 3) and used to find the center point of the caudal fins in both images just as in the original pipeline. In summary, this resulted in an approach that was considerably less computationally demanding than the original pipeline but that still offered the same accuracy in the 3D position for each matched image pair. Since BBs from different fins are more likely to have similar shapes than the segmented masks for these, the potential cost of this modification is that the chance of erroneously matching two unrelated fins may be higher than that for the original pipeline.

#### 2.4.2 Full set of ROS2 nodes

**FIGURE 4:**
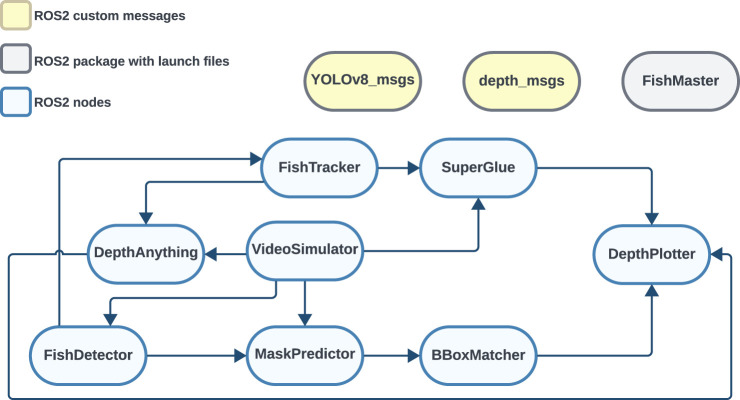
Full set of implemented nodes in ROS2.

The full ROS2 implementation (Figure 4) featured six ROS2 nodes pertaining to the three implemented pipelines, two auxiliary ROS2 nodes (*VideoSimulator* and *DepthPlotter*), one ROS2 package (*FishMaster*), and two different message formats (*YOLOv8_msgs* and *depth_msgs*). *FishMaster* contained the launch files for all pipelines, while the auxiliary node *VideoSimulator* was responsible for adapting the image stream to the method being used (i.e., providing a monocular image stream to *DepthAnything* and calibrated stereo streams to the other pipelines). The *DepthPlotter* provided visual output from each run by annotating the source images with 3D coordinates for each caudal fin identified. The *YOLOv8_msgs* and *depth_msgs* message formats defined the contents of messages describing YOLOv8 detection and the final 3D point outputs from the pipeline, respectively.

#### 2.4.3 Time synchronization

Using separate nodes to independently process the left and right frames of a stereo pair enhances parallel processing, thereby both saving time and increasing the efficiency of the pipelines. However, this requires accurate time synchronization to coordinate the node outputs, especially when these are used together in subsequent processes such as matching. It is crucial to correctly link bounding boxes and mask center points with the corresponding frame number to enable the association between the video and estimated trajectories. To achieve this, the *TimeSynchronizer* message filter, which is available as an official ROS2 package, was used. This filter synchronizes incoming channels based on the timestamps of each data point by issuing a single callback for handling the synchronized data. To facilitate this in the present system, the *VideoSimulator* node was set up to assign identical timestamps to both left and right frames of each stereo image pair before they were used in further processing in other modules (i.e., *FishDetector*, *FishTracker*, and *MaskPredictor*).

### 2.5 Method evaluation, comparison, and validation with field data

When the pipelines were ready, they were subjected to a series of tests to evaluate their performance. All tests were based on footage obtained during the field study presented by Zhang et al. (2024). The pipelines were set up to find the relative distance to all detected and tracked individuals in each frame and then output these as datasets of 3D positions and IDs. To properly benchmark the pipelines, they were subjected to tests exploring their depth estimation abilities, validating their outputs against sonar data, and scrutinizing their internal properties and abilities. The first set of tests explored their abilities in depth estimation and trajectory tracking, the second set of tests compared the estimated distances with sonar data from the field study, and the third set of tests were set up to compare the properties of the methods in more detail.

#### 2.5.1 Depth estimation and trajectory tracking

Since all methods used the computed distances to individual fish in each frame as a basic metric, the first test compared the distances estimated by the pipelines for a selection of individual fish within a single frame. To investigate their tracking abilities over time, the second test entailed applying the pipelines to short (5–10 s) clips of video, where one or more individuals were clearly visible throughout the clip. For each of these clips, the individual deemed to be most visible was chosen to be the focus of the test. The 3D trajectories estimated using the different methods were then obtained by concatenating the 3D positions given out per frame for all pipelines. Given that the distance from the camera to fish was the main focus of this experiment, the trajectories were plotted as an x–y plot and a separate plot of z vs. time. Since there exists no ground truth for these cases, the comparison could only be done visually and qualitatively.

Since *DepthAnything* is monocular and hence only needs one image stream, it was subjected to an additional test to better illuminate the potential (and limitations) of this method for future applications. This test was focused on the fact that the metric distance returned by *DepthAnything* relies strongly on the weighting applied to the relative depth map. It was thus interesting to investigate the impact of applying a different weighting on the distance estimates obtained with this method. Another instance of *DepthAnything* pre-trained using the KITTI dataset (which is based on outdoors images) was, therefore, downloaded and compared with the version trained using NYU-Depth v2.

#### 2.5.2 Distance to the intrusive object

To test the pipelines against the sonar data from Zhang et al. (2024), they were set to process the videos used by Alvheim et al. (In review) that were recorded when the fish were exposed to the six different shape, size, and color combination cases. Since the aim was to scope the shortest distance from the fish to the object, the distance to the closest 3D point was used as the output for each frame in the videos. This was then averaged across all six repetitions to yield the mean minimum distance ± standard deviations for each case, which was then compared with the corresponding sonar data from Zhang et al. (2024).

#### 2.5.3 Object detection, stereo matching, and processing efficiency

To evaluate how well the pipelines could capture all relevant objects (i.e., fish) in the images, all objects detected and tracked using each pipeline in all distance to intrusive object tests were summed for each separate case (i.e., SW, SY, BW, BY, CW, and CY). This resulted in a cumulative count of the number of objects each pipeline could detect through all videos, with the expectation that higher detection rates would imply a better ability to capture the true mean and variability in vehicle–fish distances. In turn, this indicates how well the methods could be expected to capture the variability among individuals in the population (i.e., methods capturing few fish are more at risk of experiencing bias in their output than those that capture most of the visible fish). Since all pipelines used the same *FishDetector* and *FishTracker* modules, the outcome from the *DepthAnything* pipeline was considered the baseline in this case as it, by only processing images from one image stream, should yield the highest number of detections. This was necessary as there exists no manual ground-truth data on the total number of detections across all videos. The efficacy of the stereo-based pipelines in this metric, on the other hand, depends on their ability to detect and track the same individual fish in both streams. Some variation within object detection efficiency was, therefore, expected between these.

Although all stereo-based methods found the distance by identifying key points in both video streams, they used different approaches to achieve this. Since high-quality matches are critical for accurate depth estimation, the matching quality of the stereo-based pipelines was also compared. Although there existed no ground-truth data for this comparison, qualitative considerations could be made based on how well the points in the left and right images coincided with visually recognizable features in the images.

The final element of inter-method comparison was real-time performance when implemented in ROS2 on a desktop computer. This was done by analyzing the mean update rate of the modules comprising each pipeline (i.e., *FishDetector*, *FishTracker*, *DepthAnything*, *SuperGlue*, *MaskPredictor*, and *BBoxMatcher*) after implementation in ROS2. It was assumed that an update frequency of 5–10 Hz would be sufficient for use in underwater navigation scenarios and that the methods that can operate within this interval were good candidates for real-time applications. In these tests, each pipeline was executed on three selected video recordings from the field datasets. Each video lasted 1 minute, with the initial 100 frames being excluded from analysis to ensure accuracy in computing mean processing times as this would account for the necessary warm-up period of the components and models.

## 3 Results

### 3.1 Depth estimation and trajectory tracking

#### 3.1.1 Depth estimation

**FIGURE 5:**
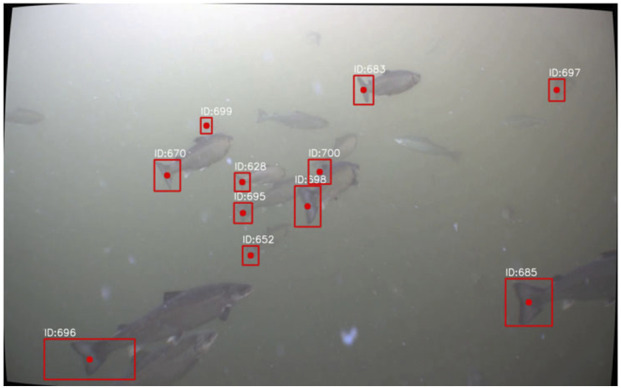
Example illustrating the detection of 11 individual fish in an image. Each individual is assigned a unique ID that is used throughout all the tracking processes.

Figure 5 shows a single frame from a video marking nine individual fish that were identified by all pipelines, while Table 1 contains the distance estimates of all methods to all nine individuals, ranking these from the closest (superscript ^1^) to the furthest away from the camera (superscript ^9^). The distances estimated for the individuals using the three stereo-based methods were similar, albeit not identical. Although all three pipelines assessed IDs 628 and 695 to be the furthest from the camera, *SuperGlue* assessed fish 695 to be furthest away, while the others concluded on fish 628. Moreover, although all stereo methods had fish 652, 670, and 699 ranked at distances 5–7 from the camera, *BBoxDepth* had a different order of these (670, 652, and 699) than the other two (652, 699, and 670). Aside from this, the methods were in consensus, and in particular, all individual rankings made by *SegmentDepth* were in agreement with at least one of the other methods. *DepthAnything* provided values that differed more from those obtained using the other methods. This also led to a distance-based ranking of the individuals that differed from the rankings generated by the other methods in all slots, with the only common element being that fish 685 and 696 were identified as the two closest to the camera.

**TABLE 1:** Estimated depth of all detected individuals in Figure 5 using the four pipelines. For each pipeline, superscripts denote the ranking of the individuals from the closest (superscript^1^) to the furthest away (superscript^9^).

ID	SuperGlue (m)	SegmentDepth (m)	BBoxDepth (m)	DepthAnything (m)
628	2.174^8^	**3.748** ^9^	**2.734** ^9^	2.302^5^
652	$1.582^{5}$	1.898^5^	1.960^6^	2.844^7^
670	$2.017^{7}$	2.015^7^	1.659^5^	1.896^3^
683	$1.350^{3}$	1.378^3^	1.414^3^	2.114^4^
685	$0.670^{1}$	$0.678^{1}$	**0.717** ^1^	1.381^2^
695	**2.244** ^9^	2.221^8^	2.262^8^	2.397^6^
696	1.062^2^	0.739^2^	1.011^2^	**1.300** ^1^
697	1.514^4^	1.471^4^	1.630^4^	2.848^8^
699	1.933^6^	1.955^6^	2.227^7^	**3.400** ^9^

The closest and furthest distances for each pipeline are also highlighted in **bold** text.

#### 3.1.2 Trajectory tracking

**FIGURE 6:**
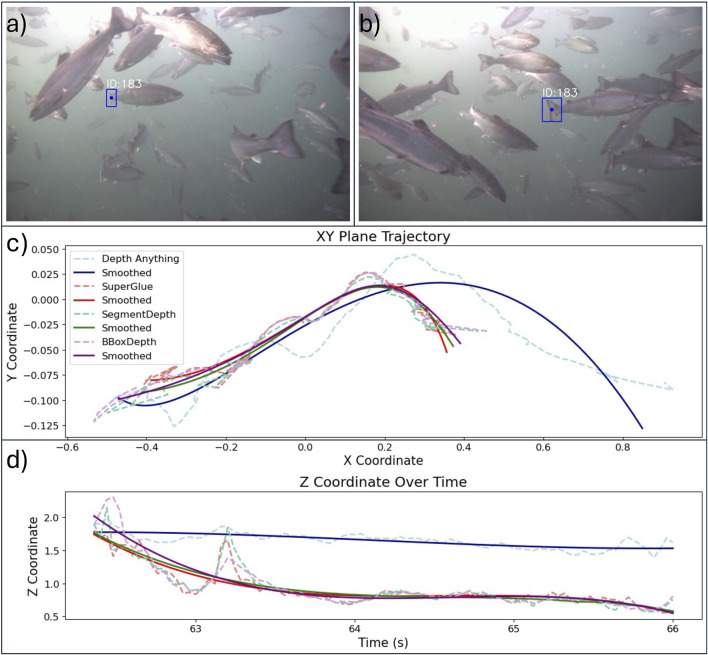
Example of caudal fin tracking for individual with ID 183. **(a)** Detection at the start of the track; **(b)** detection at the end of the track; **(c)** XY tracks of the caudal fin obtained using the different pipelines; **(d)** time series of the Z-coordinate of the caudal fin estimated using the different pipelines. All pipeline trajectories are shown using both raw and smoothed data.

The outcomes from a video segment of 5 s were used to illustrate the tracking abilities of the pipelines (Figure 6). In this video, the individual with ID 183 was chosen since it was clearly visible through the track (Figures 6a,b). Both the raw and smoothed tracks (Savitzky Golay filter) of all pipelines were used in the X–Y plot and time series for Z (Figure 6c). Despite some short-term differences, the three stereo-based methods agreed on the shape and length of the fish trajectory in this video segment. *DepthAnything* estimated a trajectory that was longer in the X–Y plane but had considerably less variation in Z.

#### 3.1.3 Alternatively trained *DepthAnything*

**FIGURE 7:**
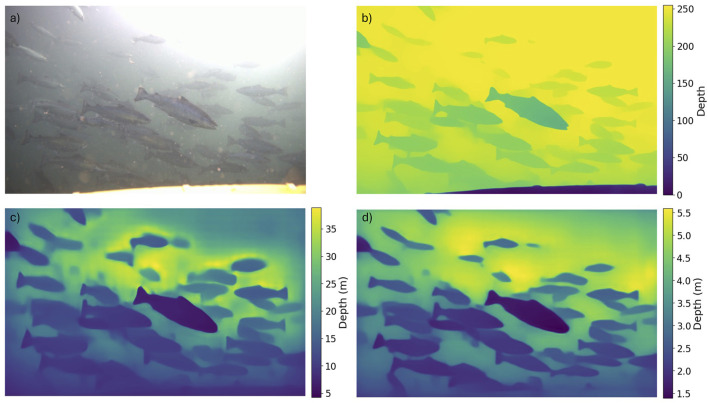
Example outputs from *DepthAnything*comparing different depth maps. **(a)**Original image; **(b)**relative depth map prediction made using *DepthAnything*; **(c)**conversion of relative depth map to metric depth map using outdoor weights; **(d)**conversion of relative depth map to metric depth map using indoor weights.

Figure 7 illustrates the impact of using different training datasets on the accuracy of *DepthAnything* in assessing metric distances. This demonstrates the transition from the original image (Figure 7a) to the relative depth map obtained using *DepthAnything* (Figure 7b) and how the resulting metrics vary when using weights based on outdoor (Figure 7c) and indoor (Figure 7d) datasets. Although the relative depth map clearly implies that the method can distinguish between differently spaced fish, the different weights resulted in vastly different estimates. When trained with the KITTI dataset, *DepthAnything* estimated distances that exceeded the expected underwater range of the camera (i.e., fish being estimated to be between 5 and 30 m from the camera). This contrasted with the version trained on the indoor dataset, which provided distances within the camera range, estimating the fish to be 1.5–5 m from the camera.

### 3.2 Distance to intrusive objects

**FIGURE 8:**
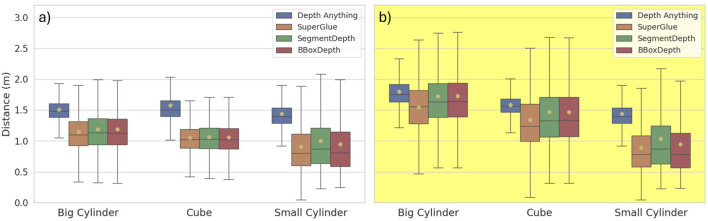
Mean depth and variability in the distance estimated using the four processing pipelines to intrusive objects of different shapes (cylinder or cube) and sizes. **(a)** Boxplots of estimated distances when the intrusive object was white; **(b)** boxplots of estimated distances when the intrusive object was yellow.

Figure 8 contain boxplots describing the mean and variability in the minimum estimated distance from the object carrying the cameras and the fish closest to this object.

The monocular method (*DepthAnything*) estimated larger distances than stereo-based methods in all cases. Furthermore, although *SuperGlue*, *SegmentDepth*, and *BBoxDepth* had similar trends across cases, *SuperGlue* tended to have slightly lower mean values than the other two. These observations are confirmed through Table 2, which provides the numerical values for the mean and variability in distance. The last column of the table describes independent reference distances obtained using a sonar by Zhang et al. (2024). Although the trends and distances obtained with sonar are similar to those obtained with the vision-based methods, in some cases, the mean values from computer vision varied between being shorter (CY) and longer (BW, SW, and SY) than the sonar-based data. Which of the four methods aligned most closely to the sonar estimate was found to vary between the cases.

**TABLE 2:** Mean ± standard deviation in m of distances between the object and the nearest fish for all computer vision pipelines used in the study (first four columns). The final column provides the corresponding findings using sonar by Zhang et al. (2024), which serves as an independent reference.

Case	Mean ± standard deviation [m]				
	*DepthAnything*	*SuperGlue*	*SegmentDepth*	*BBoxDepth*	Reference
BY	1.80 ± 0.25	1.55 ± 0.44	1.72 ± 0.53	1.73 ± 0.54	1.72 ± 0.21
BW	1.51 ± 0.21	1.15 ± 0.33	1.19 ± 0.36	1.19 ± 0.37	0.93 ± 0.06
CY	1.59 ± 0.20	1.34 ± 0.48	1.46 ± 0.59	1.47 ± 0.59	1.72 ± 0.26
CW	1.58 ± 0.30	1.05 ± 0.26	1.06 ± 0.28	1.06 ± 0.28	0.99 ± 0.04
SY	1.65 ± 0.30	1.38 ± 0.54	1.64 ± 0.79	1.64 ± 0.79	1.23 ± 0.25
SW	1.44 ± 0.27	0.91 ± 0.41	1.00 ± 0.54	0.95 ± 0.53	0.77 ± 0.05
MAE	0.412	0.187	0.205	0.197	
CI (95%)	0.148–0.676	0.073–0.300	0.050–0.360	0.046–0.348	

Abbreviations for cases: BY, big cylinder yellow; BW, big cylinder white; CY, cube yellow; CW, cube white; SY, small cylinder yellow; SW, small cylinder white. Mean absolute error (MAE) and the 95% confidence interval [CI (95%)] relative to the reference values are provided for all camera-based methods.

### 3.3 Object detection, stereo matching, and processing efficiency

#### 3.3.1 Object tracking ability

**FIGURE 9:**
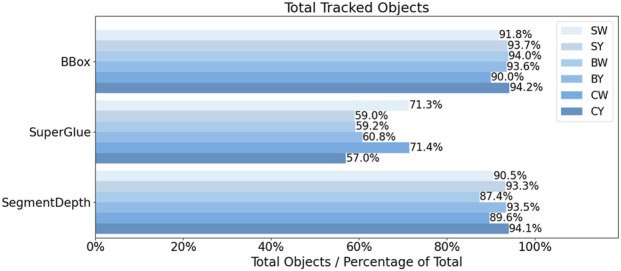
Comparison of object tracking success rates for the three stereo pipelines for each case study. The reference values for the percentages are the total numbers of objects tracked using the *DepthAnything* pipeline.

Since all methods used the *FishDetector* module for object detection, it was expected that *DepthAnything* would be able to track more objects than the others since it only needed to identify objects (i.e., fish) in the left camera stream. Although the number of detections varied between cases, the total number of detections were in the same order of magnitude (SW: 52967 detections, SY: 32855, BW: 60669, BY: 60301, SW: 25813, and CY: 60882). This number was thus considered the benchmark when evaluating the performance of the other methods (Figure 9).

The number of objects tracked using *DepthAnything* varied across cases from 25813 (CW) to above 60000 (BW, BY, and CY). *BBoxDepth* and *SegmentDepth* were comparable in consistently capturing 87%–94% of the objects tracked using *DepthAnything*, while *SuperGlue* was less successful, ranging from 57 (CY) to 71% (SW and CW).

#### 3.3.2 Quality of matches

**FIGURE 10:**
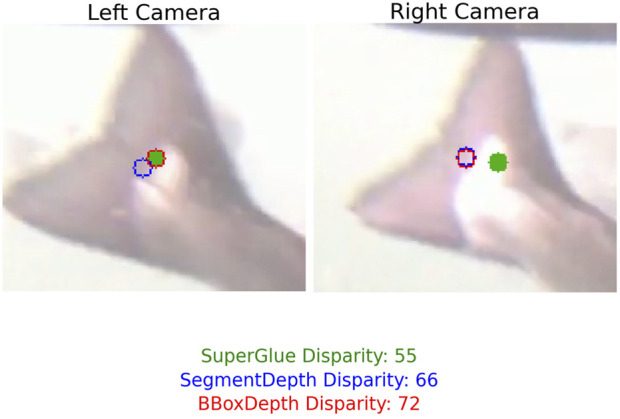
Example of matched points from *SuperGlue* (green), *SegmentDepth* (blue), and *BBoxDepth* (red).

An example of matched points used for triangulation across the three methods illustrates how the methods performed differently in stereo matching (Figure 10). In general, *SuperGlue* and *SegmentDepth* appeared better at recognizing the same features in both image streams than *BBoxDepth*.

#### 3.3.3 Real-time performance

The runtime of the nodes after implementation in ROS2 was variable, as evident from the registered publication frequency of the node outputs (Table 3). The common modules used by all pipelines (*FishDetector* and *FishTracker*) had updated frequencies above 24 Hz, which is well above the desired 10 Hz for real-time robotic operations. Of the pipeline-specific modules, *MaskPredictor*, *DepthAnything*, and *SuperGlue* all delivered update frequencies at between 6.251 and 7.845 Hz, while *BBoxMatcher* delivered update frequencies more rarely at 1.512 Hz. In summary, this led to the pipelines ranging from 4.75 Hz/0.21 s latency to 1.15 Hz/0.88 s latency.

**TABLE 3:** Publishing frequency of ROS2 nodes, including both nodes common to all pipelines (*FishDetector*and *FishTracker*), and pipeline-specific nodes.

Node	Mean rate (Hz)	Latency (s)
FishDetector	24.657	0.04
FishTracker	24.259	0.04
MaskPredictor	7.845	0.13
DepthAnything	7.774	0.13
SuperGlue	6.251	0.15
BBoxMatcher	1.512	0.67
Pipeline		
DepthAnything	4.75	0.21
SuperGlue	4.13	0.24
SegmentDepth	1.15	0.88

## 4 Discussion

This study was aimed at comparing different methods for assessing the depth in underwater images with the intent of creating a basis for the future development of robot perception systems for navigation in aquaculture net pens. The three stereo-video pipelines provided distance estimates that largely matched the sonar-based data obtained in the field study and were proven to have a potential for real-time tracking of individual fish. Although this implies that all these methods may be sufficiently accurate to be used in underwater navigation, the other evaluation metrics helped differentiate between them. First, *SegmentDepth* and *BBoxDepth* evidenced better detection rates than *SuperGlue* in detecting more of the objects of interest. Second, *BBoxDepth* is more likely to suffer from inaccurate positioning than the other methods since it triangulates using the centroid of bounding boxes rather than actual image features. In summary, these observations suggest *SegmentDepth* as the most promising candidate of the three pipelines. This impression is further strengthened by the fact that all elements in the distance-based ranking of the individuals provided by *SegmentDepth* in the initial depth estimation test were in consensus with at least one of the other methods. However, for this pipeline to also satisfy the real-time requirements of underwater navigation, the *BBoxMatcher* module in ROS2 would have to be improved such that it achieves a higher publication frequency (minimum 5 Hz) than that registered in the runtime analysis. This is particularly important considering that most robotic platforms have lower hardware capabilities and performance than the computer used in this study.

Although *DepthAnything* was not sufficiently accurate in its present state, proper training can partly address this shortcoming. With improved accuracy, this method could be considered promising for future use as it requires only a single video stream to provide distance data to the fish. This means that the method could be applied to the standard cameras typically carried by underwater vehicles, which reduces the need to invest in and install a more complex sensor package. However, the benefit of simpler instrumentation needs to be weighed against the desired accuracy as it is unlikely that any monocular method has the potential of achieving the same accuracy as stereo-based methods.

In summary, the methods presented and tested in this study will complement other relevant parallel developments. This includes studies using similar pipelines leveraging deep-learning to segment and track cell movements (Wen et al., 2021) and recent developments within underwater robot perception and navigation that enable 3D tracking based on other approaches, such as probabilistic semantic world modeling (Topini and Ridolfi, 2025).

### 4.1 Depth estimation and trajectory tracking

#### 4.1.1 Depth estimation

Although there existed no ground-truth data on depth estimation, all four pipelines provided plausible distance estimates to the fish identified in the images. Despite their common features in fish detection and tracking, the three stereo pipelines used different principles to derive the pixel-based positions. The strong similarities between their absolute distances and distance-based rankings thus indicate that all three methods were robust and accurate at assessing the depth in 2D images. *SegmentDepth* appeared to be slightly more reliable than the others since all elements in the distance-based ranking it provided were in agreement with at least one of the other pipelines. Although these metrics and features varied between images, this pattern was a consistent trend among images tested, supporting these conclusions.

*DepthAnything*, in contrast, provided estimated distances that deviated from those of the other methods. This resulted in a completely different distance-based ranking from that of the stereo-based methods, suggesting that the monocular approach was more inaccurate in assessing distances to the individual fish than these. It is likely that this perceived inaccuracy in estimates from *DepthAnything* is partly because monocular methods have inherent challenges with scale ambiguity as they have to rely on the ability to convert scales and perception into metric distances. This is further complicated by the method not being specifically trained for underwater applications and fish detection. One interesting observation was that the difference between estimates from *DepthAnything* and the other methods decreased as individual fish were farther from the camera. Moreover, *DepthAnything* appeared to show better consistency in tracking between subsequent frames than the stereo-based methods for fish far away from the camera or when the water was turbid. This suggests that this approach may have a higher robustness when the visibility is poor than that of the stereo-based methods. A possible explanation for this lies in that, unlike *DepthAnything*, the stereo-based methods depend on correctly tracking the fish in both video streams. Stereo tracking is likely to suffer when the visibility is poor since the inaccuracy and uncertainty of the stereo matching then increase.

#### 4.1.2 Trajectory tracking

For the single-frame depth estimation, all pipelines provided realistic and plausible trajectories when set to track-selected individuals in video clips. Although there existed no ground-truth data for this case, all three stereo-based methods provided similar trajectory estimates, while *DepthAnything* predicted a different path that was farther away from the camera. This was also the general trend throughout the analyses of all such trajectories, supporting the notion that the stereo-based pipelines were more accurate at depth estimation than the monocular method. Although the stereo-based methods largely agreed, there were fluctuations around their final trajectories. This is probably because stereo matching had to be applied for each frame. The process of identifying features in a single image is subject to uncertainty due to noise and inaccuracy. Requiring the detection of the same features in both images therefore introduces even larger uncertainties and variations into the tracking process.

The stereo-based methods were also susceptible to noise due to their respective approaches to stereo matching. In *SuperGlue*, the center of the BB in the left stream is found first and then used to estimate the likely position of this point relative to the tracked features in the right stream. This estimate is, thus, based on the relative position of the BB center to the matched points identified within the BB in the left image, rather than on any actual features in the image. In consequence, the accumulated impact of minor inaccuracies in the positioning of the matched points could result in noise in the final 3D position estimate and hence the trajectory. *BBoxDepth*, on the other hand, used the center points of matched BBs from both images and should, as such, have a closer link with the image contents. However, the BBs enclosing an object may be perceived slightly differently in the left and right images due to the difference in the position and attitude of the fish relative to the two cameras. This can, in turn, lead to discrepancies resulting in fluctuations in the output trajectory. *SegmentAnything* is less sensitive to such disturbances as it relies on first segmenting the caudal fin and then finding the midpoint of the mask. This approach is more closely linked with the actual image contents than the other two and is thus more likely to yield more reliable and robust results. However, in cases where the tracked fin assumes oblique angles or other poses that are suboptimal for tracking, this pipeline also exhibits variations and fluctuations, as reflected in the estimated trajectories.

*DepthAntyhing* estimated considerably smoother trajectories than the other three, which implies that it had a high confidence in its estimates. This is most likely because the method is monocular and thus is not susceptible to the challenges related to stereo methods mentioned above. The smoothness also implies the potential of this method for future use in robotics since smooth trajectories can be used to derive more consistent reference values that may, in turn, be useful as inputs to a control system.

#### 4.1.3 Alternatively trained *DepthAnything*

Although *DepthAnything* estimated realistic distances when trained using indoor data, some of the distances provided by the version trained with the KITTI dataset were unrealistically far away from the camera (fish at 5–30 m distance from the camera). This was not unexpected since the ranges of distances in the images used to build the two datasets were very different, with outdoor pictures featuring longer distances than those taken indoors. As a consequence, the training process could bias the method toward longer distances (the outdoor case) or shorter distances (the indoor case), depending on the properties of the training dataset. This underscores the limited generalizability of monocular metric depth estimation models to new depth scales and environments once they have been trained, which is a key challenge for this method.

In aggregate, the different tests run with *DepthAnything* have also illustrated some more generic challenges related to the functionality of the method. Although it appeared able to outline individual fish based on their proximity to the camera, the method could not outline the contours of each fish very well. This could be an issue in cases where the fish density is high and individuals are close, making individual recognition more difficult. Moreover, the metric depth maps were unable to accurately describe distant backgrounds in the images as being far away (i.e., extending beyond the range of the camera) and rather labeled these to be a fixed distance from the camera. This could pose a problem, particularly if the aim is to compute, for example, the average distance to nearby objects, where it would be natural to exclude the background from the computations. These limitations did not have an impact on the outcomes of the present study as the distances to the fish were found referring to the positions of the center points of the bounding boxes in the depth maps. However, improving these aspects could expand the applicability of this method to also include new dimensions such as direct identification of individual fish without preceding object detection and more accurate 3D descriptions of in-pen environments.

The most effective measure to mitigating the challenges of *DepthAnything* would be to train the method using new datasets that describe metric distances in underwater images. All existing datasets used to train the method are based on pictures taken in air and hence do not account for visual features encountered in aquatic settings such as light attenuation, distortion, and turbidity. Moreover, existing training datasets do not contain images of fish in different shapes or sizes, which can be a crucial element in accurately assessing the distances to fish based on monocular inputs. A new dataset should thus contain fish of different species, life stages, and ages across a range of environmental conditions to ensure efficient and precise training. Since it is hard for humans to assess distance directly based on images, manually labeling such a dataset would be both difficult and very time-consuming. A better approach could, therefore, be using a reliable stereo-based distance estimation method, such as RAFT-stereo or triangulation, together with ZoeDepth, to generate depth maps automatically. Although this integration would require an implementation effort, applying this approach to stereo images could generate large amounts of data for fine-tuning *DepthAnything* for more advanced underwater applications in the future.

### 4.2 Distances to intrusive objects

The outcomes of the stereo-based method were also largely in agreement with the sonar data obtained by Zhang et al. (2024), indicating that they observed the same phenomenon, albeit through a different measurement principle. This validates their accuracy in gauging vehicle–fish distances and hence their potential utility as a component in future solutions for robotic perception. In contrast, the consistently higher distance estimates from *DepthAnything* indicate that it was less accurate, corroborating the impressions from the other trials. However, the silver lining of this observation is that such a systematic overestimation could also be interpreted as a sign of consistency. This suggests that the method could, indeed, be a reliable and useful tool given proper training data.

For some scenarios, the stereo methods provided either generally longer (BW and SW) or shorter (CY) distances than the sonar. The most likely explanation for these disagreements lies in the way in which the two different principles collect data. Although the video-based methods can assess the distances to all identified fish in each picture frame, the sonar-based method collects data in the form of 360 ° sweeps horizontally around the unit. Data from one sweep thus describe the formation of the fish group surrounding the object through the entire sweep interval. To obtain more robust data, Zhang et al. (2024) derived cumulative fish presence (CFP) images, in which the observed formations from all sweeps conducted over a 1-, 5-, or 10-min period were accumulated. The CFP images were then subjected to a deep learning network trained to identify the inner perimeter of the formation of fish captured in each image. Finally, the output from the method was the shortest distance between the unit and this perimeter [see Zhang et al. (2024) for more details]. The averaging over time in this approach can mask the impacts of fish that are particularly close to the object from the sonar data. This likely explains cases where the stereo-video methods assessed shorter distances than the sonar method as the optical methods took fish close to the camera more into account in the estimates and is probably why the standard deviation of the camera-based methods was higher than that of the sonar-based method.

Conversely, these methodical differences could also provide an explanation for the cases where video-based methods estimated longer distances than the sonar. Since the sonar-based method averages the CFPs over time, it would be less able to pick up cases where the closest fish are relatively far from the device as these would effectually be filtered out due to the averaging. Such situations would have a larger impact on the outputs from the camera-based methods as these got one distinct data point per frame, indicating that cases where fish are farther from the camera would count as much as other cases. This effect would most likely be more pronounced in periods where there is low occlusion of camera images due to fish staying close to the object.

Rather than concluding that either sonar- or camera-based methods are more accurate in assessing object–fish distances, the findings in this study illustrate that the two principles have different traits and properties that should be considered complementary. Although the sonar approach seems robust at capturing static fish responses over longer time spans (e.g., how far the fish generally stay from vehicles of a certain size, color, and shape), this approach can never capture transient responses exhibited when the situation changes abruptly. However, such changes are easy to pick up with the camera-based methods since they have a much higher update frequency (in this particular scenario, up to 5–10 Hz) than the sonar (time resolution in minutes). The outcomes of this comparison thus imply that multimodal sensory setups should be used for in-pen fish-relative navigation purposes rather than being based on only one measurement principle. Recent developments have identified algorithms for safer collision-free autonomous operations in net pens containing fish and flexible structures, such as nets and other components (e.g., Amundsen et al., 2024), which need perception systems that are robust in both stationary and transient situations.

### 4.3 Detailed comparisons of methods

#### 4.3.1 Object tracking ability

Since monocular methods only need to detect objects in one of the camera streams, it was expected that *DepthAnything* should have the highest object detection rate of the four pipelines. Some loss in this metric is expected with stereo-based methods as there will be cases where a detection appears in only one video stream since the cameras are spaced and hence do not capture the exact same scene at a given time. In this study, this type of loss was relatively low since both *SegmentDepth* and *BBoxDepth* detected almost all fish identified by *DepthAnything*. *SuperGlue*, on the other hand, tracked considerably fewer of these. This is probably because it applied a different stereo matching method than the other two pipelines. Although matching in *SegmentDepth* and *BBoxDepth* is intrinsically linked with the fish, *SuperGlue* is, in practice, “domain independent” as it analyzes a stereo video stream and identifies matches regardless of the content of the images. Although this can be considered a strength since the method may then be easily adapted to new applications, it can also reduce its accuracy in specific cases. The *SuperGlue* version used in this study was trained on terrestrial datasets, and it is likely that it would perform better if also trained using data from underwater environments featuring fish, an improvement to consider for future applications.

#### 4.3.2 Quality of matches

In most applications, the reliance on stereo matching is not a major challenge in itself as a short baseline setup in air will tend to result in left and right image streams that both provide a good rendition of the motive and hence are relatively easy to match. However, a net pen is a complex cluttered underwater environment where illumination levels and turbidity may vary greatly even over short distances. In addition, since the camera is placed in the same volume as the fish, there is always a risk that fish may swim so close to the camera that they may occlude one or both of the cameras. These factors can make stereo matching more challenging, especially if the impact on the two cameras differs. In most cases with differential occlusion or turbidity, it is likely that the *BBoxDepth* and *SegmentDepth* pipelines would simply fail to get a match in the stereo matching, resulting in fewer data points. *SuperGlue* would be less sensitive to this since it stereo matches using features and not bounding boxes but be more susceptible to other errors arising due to these effects. In particular, differentiated occlusion and varying visibility could disturb the visual appearance of different features to the extent that they are perceived using the *SuperGlue* method as being the same. This could lead to large errors in the positioning.

Although the discrepancy between key points identified by *BBoxDepth* often appeared to be larger than those by the other stereo pipelines, this statement can only be considered indicative due to the lack of proper ground-truth data. However, such an effect can be explained by the differences in how the three methods acquire the key points. The approach of matching the centers of BBs from both images used by *BBoxDepth* can lead to larger deviations since BBs are only abstract entities derived from the picture rather than pertaining to the actual image features. Minor discrepancies in box size and placement could thus lead to larger errors in the 3D positioning. The other two pipelines were probably less sensitive to such effects as they related more strongly to the actual image contents by matching actual image features (*SuperGlue*) and using segmentation masks based on the image contents (*SegmentDepth*). In cases where the fish is swimming at a fixed distance from the camera (i.e., moving in parallel with the baseline spanning between the two cameras), this error is likely low since the bounding box encasing the caudal fin in both images is probably of similar size and similarly positioned relative to the fin. However, when a fish moves at an oblique angle relative to the baseline, the shape, size, and location of the BBs relative to the caudal fins will differ more from left to right. A more thorough analysis quantifying such discrepancies across matched cases could be applied to support this finding.

Finally, BB matching can also result in erroneous matches because its matching procedure depends solely on BB positions and sizes. In scenarios where the fish are kept in high densities, there will be a risk that situations where two or more fish of roughly the same size stay close to each other may occur. Due to their proximity and similar size, the BBs representing the detection of these fish would be both close to each other and similarly sized, rendering the matching of boxes from the left and right images more challenging for the method.

#### 4.3.3 Real-time performance

Although a true evaluation of the real-time performance of the pipelines cannot be done without first implementing them on a real mobile platform, the evaluations done in this study sought to provide proof of concept that the methods should be runnable in ROS2. Considering the wide variety of hardware resources available in underwater robots and that these often have limited capacity compared with desktop computers, this was thus considered an important prerequisite before future testing in real field deployments.

*FishFinder* and *FishTracker* maintained rates close to the video frame rate at 25 fps, which is well above the desired interval of 5–10 Hz. Moreover, the modules specific to *DepthAnything* and *SuperGlue* were also within this interval, implying that they too have potential to be applied in operational studies. Of these, *DepthAnything* appeared marginally more efficient probably because it is a streamlined process with little inter-process communication and depends on a single camera stream rather than two. It should here be noted that the ability to deploy and run these in the field would finally depend on the computational abilities of the chosen platform, robot, or vehicle.

The picture is more complex when considering the modified *SegmentDepth* pipeline. Although *MaskPredictor* had an even faster update frequency than *SuperGlue* and *DepthAnything*, achieving a latency of 0.13 s, the *SegmentDepth* pipeline also features *BBoxMatcher*, which had the slowest publication rate of all nodes, with a latency of 0.67 s. This is probably not related to the inference time of this node, but rather a result of synchronization issues that arise when combining complex nodes in the same pipeline. Although the *MaskPredictor* node processed images and detections from both cameras, the resulting datasets do not always have the same time stamps. *BBoxMatcher* will then struggle to synchronize and process mask matching points from both camera feeds effectively. This highlights a critical area for improvement in ensuring better synchronization and data alignment within the pipeline.

To complete the validation of these methods for field use, future studies should seek to improve the efficiency of the slowest modules to prevent them from bottlenecking data throughputs. Moreover, it is also important that future tests entail implementing the pipelines at actual underwater robots or vehicles. This would demonstrate whether the pipelines can achieve sufficient real-time performance when all modules are run together and whether they can be run efficiently on the hardware used to run such devices. Additional insight can also be gained by testing the pipelines on several different embedded platforms since they may vary greatly in hardware components and hence processing power. This would shed light on their performance when run on different systems, which will be an important step before introducing them for active use with actual underwater robots.

### 4.4 Future applications in underwater vehicles

#### 4.4.1 Pipeline improvements

Although the pipelines tested in this study performed well at the intended tasks, they would need some further refinement and improvements to be fully applicable as industrial tools for underwater navigation in net pens. One important aspect is robustness with respect to factors that may complicate fish detection and tracking. In their present state, the methods have been trained using footage collected when visibility and lighting conditions were good, providing good contrast between the fish and the background. This would not always be the case during robotic operations as the natural light level in salmon net pens varies greatly during the production cycle due to the annual (e.g., winter vs. summer) and diurnal (e.g., dusk and dawn vs. noon) variations and with depth. Moreover, feces and particular waste from feed may, at times, make the water much more turbid than in the footage used in this study. An important step in improving the robustness of the pipelines would, therefore, be to also train these using videos collected during darker periods and with high turbidity. These new data should complement the existing datasets to ensure that the resulting method is effective at detecting fish under both beneficial and sub-optimal visibility conditions.

Another factor that is important to consider is that video footage of fish swimming in dense shoals often results in a greater degree of overlap and occlusion between individuals in the images than when the fish are more sparsely distributed. This may render the segmentation, feature extraction, and tracking of individual fish more difficult as the bounding box containing the detected object also contains parts of the background, which are composed by other fish that may complicate segmentation and extraction. The choice of focusing on caudal fins instead of whole fish bodies in this study reduces this complexity as the boxes used in the detection are smaller and thus contain fewer proportions of other fish, thereby reducing the risk of erroneous detections or segmentations. However, this effect is probably not sufficient to fully compensate for this source of inaccuracy. One possible way of improving this could be using oriented bounding boxes (OBBs), which is an improvement where the bounding boxes are oriented to fit better with the contours of the detected objects, thereby circumscribing the object more closely than when the bounding box is horizontally oriented. OBBs have previously been used for different applications, including tracking of the motion and orientation of farmed pigs (Liu et al., 2023). In other animals, it is likely that the use of OBB would improve the performance of fish tracking as the bounding boxes would then be more constricted around the object of interest (in this case, caudal fins) and thus include less background that may contain parts of other fish.

#### 4.4.2 Vehicle implementation and field deployment

This research was motivated from a perspective of enabling future underwater vehicles to minimize their impact on the fish while conducting operations in commercial fish farms. The end-point application of the pipelines would thus be to serve as a sensory mode for the vehicles to automatically detect and track fish in the vicinity. To realize this, the observations from the pipelines would have to be used as inputs to the control algorithm of the vehicle. This algorithm could be designed such that the vehicle adapts its positioning and motion in response to the presence and movement of the fish. For instance, the position could be controlled such that the vehicle never gets within a certain distance of detected fish to prevent intruding upon them, which may elicit a startled response. Likewise, observed fish movements could also be used to control the vehicle movement speed, in that it could reduce speed when observing that fish are actively swimming away from it. This could be realized using existing motion planners with collision avoidance, such as Amundsen et al. (2024), and considering the fish as being dynamic obstacles with a certain radius. The parameters of the chosen collision avoidance method could then be calibrated to provide the proper responses toward the fish presence and movement. However, it should be noted that a fish-relative motion planner solely based on cameras would be very sensitive to visibility conditions. Although some of the improvements mentioned above will partly address this, the pipelines are not efficient if light levels are too low. This indicates that a final version of such a fish-related navigation system should also feature other modes of observation, such as sonars and other acoustic devices.

In conclusion, we believe that the pipelines tested in this study could be components in future control systems for underwater vehicles designed for in-pen operations in fish farms. If they could enable the vehicles to adjust their motions to the presence of the fish, it would be possible for them to move such that they disturb the fish less, thereby possibly reducing the potential of impairing fish welfare under production. This could have a particular impact on some of the continual and periodic tasks conducted at commercial fish farms such as net inspection and cleaning (Kelasidi and Svendsen, 2022). Due to their pervasiveness in fish farm operations, these operations have been suggested for automation to alleviate the need for manpower and manual work (Fu et al., 2024). Continuous operation using the present pipelines would put even stricter requirements on their accuracy and real-time properties as the vehicle would then have to be equipped to handle unforeseen situations and react quickly to changes. This would also entail periodically recalibrating the cameras, possibly with a permanently installed reference near the docking station of the vehicle. In addition to these technical aspects, realizing autonomous operations in commercial fish farms would require regulatory changes. These changes would likely include a set of requirements and rules for the design and operation of the vehicle to prevent unwanted interactions between the vehicle and the fish farm structures.

## Data Availability

The data analyzed in this study are subject to the following licenses/restrictions: The data are not open to share as they do not solely belong to us. Requests to access these datasets should be directed to eleni.kelasidi@ntnu.no.
